# Truth or fallacy? Three hour wait for three minutes with the doctor: Findings from a private clinic in rural Japan

**DOI:** 10.1186/1447-056X-9-11

**Published:** 2010-11-23

**Authors:** Adam N Wooldridge, Nóra Arató, Ananda Sen, Masaki Amenomori, Michael D Fetters

**Affiliations:** 1 The Ohio State University, College of Medicine, 370 West 9th Avenue, Columbus, OH 43210, USA; 2 University of Michigan, Department of Family Medicine, 1018 Fuller St, Ann Arbor, MI 48104-1213, USA; 3 Yuge Medical Clinic, 1825 Yuge, Ryuou-cho, Gamou-gun, Shiga-ken, Japan 520-250

## Abstract

**Introduction:**

While previous reports examine various aspects of Family Medicine in Japan, there is sparse research on consultation lengths. A common phrase permeates throughout Japan, *sanjikan machi, sanpun shinsatsu *that means, "Three hour wait, three minute visit." The purpose of this study is to examine consultation length in Japan, and how it is affected by patient variables.

**Case Description:**

We conducted a case study of consultation length and how it varies in relation to the demographics, presenting illness, and diagnoses at a rural clinic in central Japan. Data were coded according to the standards of the International Classification of Primary Care. Descriptive statistics were obtained to identify features of the data. Further, regression analysis was performed to characterize and to quantify the association between length of consultation and various subject level characteristics.

**Discussion and Evaluation:**

A total of 263 patients aged 0 - 93 years old had consultations during the 8-day study period. The mean consultation duration was 6.12 minutes. Of all consultations, 11.8% lasted 3 minutes or less. The mean (median) consultation time among males was 6.29 (5.2) minutes and among females was 6.03 (5.4) minutes. The duration of visits increased with age. Among different International Classification of Primary Care categories, psychological issues required the most time (mean = 10.75 min, median = 10.9 min) while urological issues required the least (mean = 5.08 min, median = 4.9 min). The majority of cases seen in the clinic were stable, chronic conditions and required shorter consultation times.

**Conclusions:**

While the mean and median consultation length in this study extends beyond the anecdotal three minutes, the average length of consultation is still remarkably short. Trends affecting consultation length were similar to other international studies. These data present only one aspect of primary care delivery in Japan. To better understand the significance of consultation length relative to the delivery of primary care, future research should examine issues such as continuity, frequency of consultations over time and comprehensiveness of care.

## Background

Japanese people live longer than any other population in the world, and neonatal and infant mortality rates are among the lowest in the world[1]. The population of Japan has universal coverage either through employee insurance or through the National Health Insurance scheme[2-5]. Japan also has a Long Term Care Insurance (*Kaigo Hoken*) program that covers disability as a consequence of medical conditions[6,7]. While the Japanese government does not recognize family medicine as a specialty in Japan, there is a national movement to develop family medicine training. Current Japanese private practitioners (*kaigyoi*) mostly are hybrid specialty care/primary care providers who trained and practiced specialty care in a hospital for 5-10 years prior to going into private practice[8]. There are 277,927 physicians in Japan with 95,213 physicians working in ambulatory clinics. Of these 95,213 physicians, 74.8 percent own a solo practice and 25.2 percent work in a group practice[9]. Since they do not have hospital privileges they provide ambulatory-based chronic and acute care, and often home care[10].

Public insurance benefits do not include preventive care that private practitioners can bill for unless there is a contract or voucher system with the local government. Most private practitioners do not provide women's health unless they trained in obstetrics and gynecology.

Prescribing patterns are a unique feature of Japanese health care. Private practitioners spend much of their day in short visits prescribing and in some places, dispensing medicines. While the legal limits of the duration of most drug prescriptions officially increased from a maximum of 90 days to more than 90 days in 2002,[11] the vast majority of physicians prescribe about a 14 day supply[12]. Recent data suggests the longest mean duration of drug dispensing from outpatient offices is 30 days with many chronic medications dispensed for less than 20 days[12]. The chronically ill make short clinic visits every 14-30 days just for medications.

Prescribing patterns strongly contribute to the most striking feature of Japanese ambulatory care practice, e.g., the number of patient consultations in a routine workday. Japanese private practitioners often see 60-100 patients a day[13]. This pattern raises questions about what is being conducted differently and the implications of how primary care is delivered. A common phrase in Japan, *sanjikan machi, sanpun shinsatsu*, meaning, "three hour wait, three minute visit" alludes to the public's less than enthusiastic opinion of the system[14].

While a smattering of articles in English address other aspects of family medicine in Japan,[15,16] there are few reports on the details of clinical practice[17]. The most informative cross-national comparative research results can be found in studies led by Okkes and Yamada[18,19]. The Okkes study collected data from several countries including Japan regarding reasons of encounters, diagnoses, and interventions[18]. However, it does not provide moment-by-moment details for a fine-grained understanding of time utilization. Other than studies of geriatric clinics and a small linguistics study of ten patient-physician interactions [14,15,17] the literature lacks empirical studies on primary care consultation length in Japan. European research illustrates that consultation length increases if the patient is older, if the patient is a female, if the reason for the encounter is a psychosocial problem, and if the clinic is in an urban setting[20]. Given this gap in the literature, the purpose of this study was to examine duration of visits and how these differ by factors such as age and gender in Japan.

## Methods

### Design

We conducted a detailed case study of primary care delivery for all patient visits during 8 days at a Japanese family physician's clinic in rural Japan. This research was approved by the University of Michigan Institutional Review Board, ID HUM00019913, and the need for written consent for adults and assent for children was waived because the research presented no more than minimal risk, assent was not practicable, and waiver would not impact the rights and welfare of subjects.

### Setting

The setting was Yuge Medical Clinic in the village of Ryuo, Japan in June 2008. Ryuo is located about 44 km east of the major city of Kyoto in central Japan. It is a town of approximately 13,000 people and its local economy is predominantly agriculturally based.

### Study Population

All patients presenting to a Japanese physician during eight consecutive clinical days in June 2008 served as the study population. Like many office-based physicians in Japan, this physician completed internal medicine training in Japan, but is a self-taught, self-declared practitionaer of family/general medicine[8]. His office hours, billing procedures, staffing and mix of outpatient care and home visits are typical to private practitioners in rural Japan. The only dissimilarity to other Japanese physicians is that he has sufficient space in his building to allow a small number of other physicians to practice part-time in his office. Otherwise, this practice is very similar to other rural Japanese clinics. In his clinic, he documents the visit in an electronic health record during the patient consultation.

### Data Collection

The observer (ANW) positioned himself in a non-obtrusive way in the consultation room. The physician introducing himself signaled the start time and extending his salutations to the patient marked the end of the consultation. The consultation length was recorded using a small digital timer to avoid disrupting the physician-patient interaction. Although the physician was aware that data about his consultation lengths would be recorded, he did not know the days that time data would be collected. Consultations were recorded in seconds, and later converted to minutes. Data on the patients' age, gender, reason for encounter, and diagnoses were recorded. The latter were later coded according to the standards of the International Classification of Primary Care (ICPC). The ICPC is a classification system useful for primary care encounters as it accounts for the reason for encounter, the problems/diagnoses, primary care interventions, and ordering of primary care data from an encounter as an episode of care[21].

### Data Entry and Analysis

Data were entered into Microsoft Excel and exported to Statistical Package for the Social Sciences (SPSS) to calculate statistics. We examined variation by gender, age, and number of diagnoses. Averages and ranges of consultation times were also determined for each of the general ICPC categories by gender. To determine time spent according to consultation type - whether acute, chronic or preventive - the reason of the visit and the diagnosis were reviewed and were coded per visit. We used the following criteria to categorize the visit types: a) acute illness visit is a condition with either a rapid onset or a short course or both, b) chronic illness visit is due to a condition that has a long-lasting course or is recurrent, and c) preventive care visit is one to prevent an illness or an injury, rather than to cure it. If a patient presented with both acute and chronic issues, he/she was categorized as having an acute problem.

Regression analysis was performed with log minutes as outcome; the variables sex and diagnosis code as factors and age as covariate. Logarithm of consultation time (in minutes) is used as an outcome since the resultant model provided a better fit to the normality assumption. Post-hoc comparisons between the diagnostic codes were carried out using Bonferroni adjustment for multiple comparisons. A two-sample t-test was used to identify any possible differences in mean consulting time between acute and chronic reasons for encounter. Further, a logistic regression analysis was carried out to identify any difference in the likelihood for acute and chronic reasons for encounter by gender and age.

The data for this study was collected from a rural, community-based private clinic similar in most respects to other private clinics in Japan. The purpose was to obtain descriptive summaries of practice patterns. Consequently, the sample size was not targeted to achieve a pre-specified power for subgroup comparisons. However, our ultimate sample size of 263 subjects was adequate to provide descriptive summaries with a sufficient level of confidence. For example, assuming the standard deviation of the consultation length to be 3.2 minutes, as estimated from our data, the true mean consultation length can be estimated to within 0.4 minutes with 95% confidence. Similarly, estimates of proportion of categorical outcomes can also be obtained with reasonable precision. For example, the true proportion of acute visit types can be assessed to within five percentage points with 95% confidence assuming the expected proportion to be around 75%, as estimated from our data.

## Results

### Demographics

The sample includes 263 patients, 174 (66%) females and 89 (34%) males with their ages ranging from 0 - 93 years (Table 1). Patients aged 65 years and older constituted 57% of the total sample. The 19-64 age range included 30% of the entire female and 40% of the male populations, respectively. Still, there were proportionately more females than males in this age group. The majority of patients (76%), received one diagnosis while 22% received two diagnoses, and 2% received three diagnoses. There was virtually no difference in the distribution of the number of diagnoses between male and female patients.

**Table 1:** Patient demographics

	Females N = 174		Males N = 89		All N = 263	
	
Characteristic	n	%	n	%	n	%
Age in years						

0-12	7	4	8	9	15	6

13-18	7	4	1	1	8	3

19-49	16	9	11	12	27	10

50-64	37	21	25	28	62	24

65-74	51	29	23	26	74	28

75-84	45	26	21	24	66	25

≥85	11	6	-	-	11	4

Number of diagnoses						

one	131	75	68	76	199	76

two	40	23	18	20	58	22

three	3	2	3	3	6	2

						

	Mean	SD	Mean	SD	P value	

Age	62.9	21.3	58.7	22.2	0.13	

### Consultation length by gender and age

The average consultation length for the entire population was 6.12 minutes. Males had a longer average consultation length (6.29 minutes) than females (6.03 minutes), although the difference was not statistically significant. Based on the multiple regression model with age, gender and diagnosis code as covariates, there is a significant positive association between consultation time and age with every 10 year increase in age corresponding to a 5% increase in mean consultation time (p < 0.001). Among the advancing age categories from 0 through ≥74 (0-18, 19-49, 50-74 and ≥74) there are 63, 23, and 17 seconds differences between categories, respectively. The consultation duration was the longest for women in the 19-49 years age group. There is a noticeable, although not a fixed increase in consultation time in higher age brackets (Table 2).

**Table 2:** Mean Consultation Length in Minutes by Age and Gender

	Females N = 174	Males N = 89	All N = 263
	
Variable	Mean Median (95% CI)	Mean Median (95% CI)	Mean Median (95% CI)
Overall	6.0 5.4 (5.6, 6.5)	6.3 5.2 (5.6,7)	6.1 5.3 (5.7,6.5)

Age			
0-18 years of age	4.6 4.5 (3.3,5.9),	5.3 5.1 (3.8,6.8)	4.6 4.5 (3.3,5.9)

19-49 years of age	7.2 5.4 (3.9,10.5)	4.6 3.8 (2.5,6.7)	7.2 5.4 (3.9,10.5)

50-74 years of age	5.7 5.1 (5.2,6.3)	6.5 5.2 (5.5,7.6)	5.7 5.1 (5.2,6.3)

≥74 years of age	6.6 6.7 (6,7.3)	6.9 5.6 (5.4,8.5)	6.6 6.7 (6,7.3)

### Consultation Length by ICPC Category

**Figure 1:**
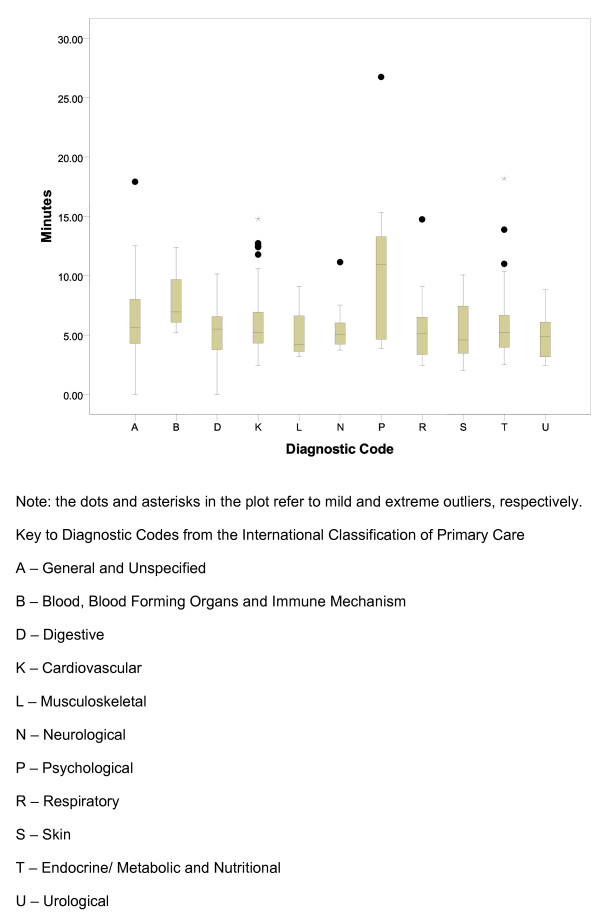
**Illness Category and Duration of Consultation in Minutes Using Boxplot**.

The longest average consultation length among the different ICPC categories was 10.75 minutes for psychological issues while the shortest average consultation length was 5.08 minutes for urological problems (Table 3, Figure 1). Males had longer consultation times in the categories of psychological, urological and respiratory issues. There is a significant difference in mean consultation time across the different diagnosis codes (P = .003). A post-hoc analysis manifested that the mean consultation time for psychological diagnosis was significantly higher than that for cardiovascular, respiratory and endocrine related diagnoses (all P-values <.05). The consultation time for psychological diagnoses was also mildly higher than skin-related diagnoses (P = .053), as well as digestive and musculoskeletal diagnoses (P = .07 for both). No other pairs of diagnoses differed significantly with regards to average consultation time.

**Table 3:** Mean Consultation Length in Minutes by Major Disease Category and Gender

	Female	Male	All
	
ICPC Category	Mean Median (95% CI)	Mean Median (95% CI)	Mean Median (95% CI)
General	5.7 5.6 (4,7.4)	7.4 5.7 (4.8,10)	6.5 5.6 (5,7.9)

Blood/Immune	8.8 8.8 (-37.1, 54.6)	*one case	8.2 6.9 (-1.2,17.5)

Digestive	5.6 5.7 (4.5,6.8)	4.1 5.3 (0.3,8.5)	5.4 5.5 (6.4,5.4)

Cardiovascular	5.8 5.3 (5.3,6.4)	6.2 5.1 (5.2,7.1)	5.9 5.2 (5.5,6.4)

Musculoskeletal	5.4 4 (2.5,8.3)	*one case	5.3 4.2 (2.9,7.6)

Neurological	6 5.2 (4,8)	4.7 4.3 (2.6,6.8)	5.7 5 (4.2,7)

Psychological	9.9 8.3 (4.2,15.5)	13.4 13.6 (8.3,18.5)	10.8 10.9 (6.6, 14.9)

Respiratory	5.2 5.1 (3.9,6.5)	7.3 5.5 (-0.9,15.4)	5.7 5.1 (4.2,7.3)

Skin	5.8 5.4 (2.4,9.2)	4.9 4.6 (-1.1,10.9)	5.5 4.6 (3.3,7.7)

Endocrine /Metabolic	6.5 5.9 (5.2,7.9)	5.6 4.7 (4.1,7.1)	6 5.2 (5,6.9)

Urological	3.7 3.7 (-12,19.3)	6 6.1 (-1,13.1)	5.1 4.9 (1.9,8.2)

### Consultation Length by Acute and Chronic Reasons of Encounter

Overall the mean consultation time did not differ significantly by visit type The majority of cases at Yuge Clinic are chronic in nature followed by acute and preventive care. Between males and females there seems to be some differences in the 19-49 and the 75-84 age groups, both in cases of acute and chronic diseases (4.05 and 2.46 minutes and 2.27 and 1.89 minutes in respective groups (Table 4). Only the last figure (1.89) signifies a difference in increase for the chronically diseased male population of 75-84 years of age. There is no difference in the likelihood for acute and chronic reasons for encounter identified by gender and age.

**Table 4:** Distribution of Acute and Chronic Visit Types with Mean Duration of Consultation in Minutes by Gender

	Female N = 39		Male N = 27		All N = 66	
	
Visit Type	n	Mean Median (95% CI)	n	Mean Median (95% CI)	n	Mean Median (95% CI)
Acute						

0-12	2	6.1 6.1 (-0.4, 12.7)	2	6.5 6.5 (-10.3, 23.2)	4	6.3 6.1 (4.4,8.2)

13-18	2	5.3 5.3 (-10.8, 21.4)	0	*no valid cases	2	5.3 5.3 (-10.8, 21.4)

19-49	5	7.6 6.4 (2.9,12.2)	2	3.5 3.5 (-0.2,7.2)	7	6.4 5.2 (3,9.8)

50-64	5	5.3 4.6 (1.6,9.1)	9	6.2 5.1 (4.1,8.3)	14	5.9 4.8 (4.3,7.5)

65-74	11	5.4 4.9 (3.9,7)	6	5 5.1 (4.2,5.8)	17	5.3 5.1 (4.3,6.2)

75-84	13	7.6 7.2 (6,9.3)	8	5.2 4.5 (3.3,7)	21	6.7 6.8 (5.4,8)

≥85	1	** one case	0	*no valid cases	1	-

Chronic	**Females N = 133**		**Males N = 58**		**All N = 191**	

0-12	5	4.1 4.5 (3.1,5.1)	6	4.3 4.1 (3.2,5.4)	11	4.2 4.3 (3.6,4.8)

13-18	5	4.3 2.7 (-0.2,8.7)	1	**one case	6	5 3.9 (1.2,8.9)

19-49	11	7.1 5.1 (2.3,11.9)	9	4.8 4.5 (2.2,7.4)	20	6.1 4.9 (3.4,8.8)

50-64	32	6 4.9 (4.8,7.2)	15	7.2 5.5 (4.5,9.9)	47	6.4 4.9 (5.2,7.5)

65-74	39	5.7 5.4 (5,6.4)	14	6.8 5.5 (4.9,8.7)	53	6 5.4 (5.3,6.7)

75-84	31	6.3 6.3 (5.5,7)	13	8.1 7 (5.8,10.5)	44	6.8 6.4 (5.9,7.7)

≥85	10	5.8 4.8 (4,7.5)	0	**no valid cases	10	5.8 4.8 (4,7.5)

**Acute illness**: an acute disease is a disease with either or both of a) rapid onset and b) short course.

**Chronic illness**: a **chronic disease **is a disease that is long lasting or recurrent. The term *chronic *describes the course of the disease, or its rate of onset and development.

### Quickest and Longest Consultation Times

Consultation lengths ranged from 1.10 minutes to 26.75 minutes (Figure 1). The three shortest consultations were for patients for preventive immunizations, dermatitis, and sinusitis, 1.10, 2.02, and 2.40 minutes respectively (Table 5). The three longest consultations were for patients with liver disease, diabetes mellitus, and depressive disorder, 17.92, 18.18, and 26.75 minutes respectively.

**Table 5:** Shortest and Longest Consultation Times by Reason for Encounter and Diagnosis*

ICPC Code	Reason for Consultation	ICPC Code	Diagnosis	Time (mins)
15 Shortest Consultations				

A44	Preventive immunizations/Medications	A44	Preventive immunizations/Medications	1.10

S06	Rash localized	S87	Dermatitis/atopic eczema	2.02

R63	Follow-up	R75	Sinusitis acute/chronic	2.40

K63	Follow-up	K86	Hypertension uncomplicated	2.43

K63	Follow-up	K86	Hypertension	2.43

U36	Follow-up	U90	Proteinuria	2.43

T63	Follow-up	T93	Lipid disorder	2.48

K63	Follow-up	K86	Hypertension uncomplicated	2.62

T63	Follow-up	T81	Goiter	2.62

S06	Rash localized	S88	Dermatitis contact/allergic	2.65

T27	Fear of endocrine/metabolic disorder	T81	Goiter	2.65

K63	Follow-up	K86	Hypertension uncomplicated	2.72

A03, R05, R08	Fever, Cough, Nose symptom/complaint	R75	Sinusitis	2.73

D12	Constipation	D12	Constipation	2.75

R05, R08	Cough, Nose symptom/complaint	R74	Upper respiratory infection acute	2.82

15 Longest Consultations				

B27	Fear blood/lymph disease	A97	No disease	12.40

K63, N63	Follow-up	K86, N87	Hypertension uncomplicated, Parkinsonism	12.42

A05	Feeling ill	Z02	Food/Water Problem	12.52

K63	Follow-up	K86	Hypertension uncomplicated	12.52

K63, N63	Follow-up	K86, N92	Hypertension uncomplicated, Trigeminal neuralgia	12.73

P63, T63	Follow-up	P70, T90	Dementia, Diabetes non-insulin dependent	12.90

P63	Follow-up	P76	Depressive disorder	13.03

P63, T63	Follow-up	P70, T90	Dementia, Diabetes	13.55

T63	Follow-up	T90	Diabetes non-insulin dependent	13.88

R05, R25	Cough, Sputum	R81	Pneumonia	14.75

K63, P63	Follow-up	K86, P76	Hypertension uncomplicated, Depressive disorder	14.80

P63	Follow-up	P76	Depressive disorder	15.33

A05	Feeling ill	D97	Liver disease	17.92

T63	Follow-up	T90	Diabetes non-insulin dependent	18.18

P63	Follow-up	P76	Depressive disorder	26.75

*Table 5 reflects single encounters that had the shortest and longest consultation lengths.

## Discussion

As to the truth or fallacy to the common perception that consultation time is only three minutes in Japan,[22] in this clinic it appears to be both. Among 263 patient consultations of one physician, the mean duration at 6.12 minutes is more than double the three-minute mark. For 31 patients (11.8%), however, the consultation actually took 3 minutes or less. If the three-minute rule has validity, the question would be why the duration in this study wasn't shorter? One factor could be recent legislative changes implemented by the Ministry of Health, Labour and Welfare (MHLW). Under this 2008 law, physicians can bill for *kanri-ryo*, a fee twice the repeat consultation fee, if the site is a clinic, the patient has a chronic condition, and the consultation length is at least five minutes (written communication, Naoki Ikegami 2/21/2010). MHLW financial incentives for extended prescription durations[11] plausibly could have an effect through decreased visits and more time per patient. Current evidence suggests the duration of prescriptions continues to be less than 20-30 days for the most common drugs[12].

While the three-minute rule is a common colloquialism, there are sparse data for comparison *within *Japan. Ishikawa et al found consultation length to average 10.5 minutes in a Tokyo geriatric clinic and a small linguistics study of ten physician-patient interactions by Ohtaki et al found consultation lengths to be 8.4 minutes, compared to only 6.19 minutes when age-adjusted in the current study[17,23]. A number of reasons could explain this intra-Japan difference, e.g., variations in the patient population with rural patients needing to be more healthy to make it to the clinic, more frequent visits and shorter consultation by rural patients, more financial pressures for productivity in the private rural office, or more patient demand for visits that would result in more time pressures in the rural clinic.

For an international audience accustomed to much longer visits, the glaring question is why would consultation length be so short? Interestingly, this mean time is approximately two and half times less than the average U.S. consultation length of 16.3 minutes[24]. While our study was not designed to assess *why *physicians can see such volumes of patients in Japan, there are several observations to consider. First, the low patient co-pay under the National Health Insurance scheme makes access easy and encourages frequent physician visits in Japan. Access facilitates continuity if the patient sees the same physician.

Second, the National Ministry of Health, Labour and Welfare, determines what compensation physicians receive for visits and procedures. The government-determined compensation fee schedule incentivizes physicians to see as many patients as possible for a short duration and on a frequent basis. Although compensation for first time visits is approximately three times that of a repeat visit, more repeat visits can fit into the schedule than first time visits. The absence of a refill system, and the lack of significant financial incentives to give chronic medications for more than several weeks results in many visits for chronic medication prescriptions[12]. Since reimbursement is based on a straightforward point system, without an option based on time as the billing system in use in the US, there would be no financial incentive to have a longer consultation. Still, frequent visits may allow Japanese physicians to be more aware of and manage early minor changes in patients' chronic illnesses.

Regarding variation in relative duration for sub-groups, these findings are consistent for the most part, with Deveugele et al's work in European countries illustrating that consultation length increases if the patient is older or if the reason for encounter is a psychosocial problem[20]. Regarding the longer consultations in urban versus rural settings in the Deveugele et al study, our data from a rural setting compared to the data collected in an urban setting by Ishikawa et al [23] are consistent with longer consultations in urban versus rural settings.

In contrast to Deveugele et al's findings on gender differences, we found consultations with men to be longer in absolute time, but not at statistical significance, than consultations with women[20]. A number of factors could account for this, but it is speculative. As men are more likely to be employed than women, it is possible that they make fewer visits, and there may be more ground to cover in a single visit. As a high percentage of patients in the study were elderly, and women tend to be healthier and live longer than men, it is also possible that the visits with men were more complex than the women. Furthermore, it is possible that gender differences are such that male patients may feel more comfortable at asking questions or challenging physician requests than women, or yet that communication patterns with male patients take longer. Further work to examine this issue is needed.

These stark differences in consultation length also raise questions about whether and, if so, how primary care works differently in Japan? While it is tempting to say that longer visits are better, this is debatable. Patients' perceptions of consultation length can be distorted--they may perceive it as shorter than actually,[25] or longer than actually[26]. Quality of time appears to be as important as the quantity of time for improving the doctor-patient relationship[27]. The three-minute colloquialism supports the perception that patients have little time with doctors *per visit*, but it may be little different per year than the US. Assuming patients with chronic medical problems see the doctor once per month, in Japan, and the average time is ten minutes, physician-patient, face-to-face time totals 120 minutes annually. A patient in the US with a chronic illness seen every six weeks (eight visits per year) for 15 minutes a visit, would be seen for about 120 minutes. This illustrates patients have significant face-to-face time with physicians, and that shorter visits in Japan do not necessarily mean patients spend substantively less time in consultations with the doctor. Whether frequent-short visits, or less frequent-long visits are better for effective delivery of primary care is uncertain.

This study has limitations. The design did not allow us to examine the duration of patient waiting, so we cannot comment on the veracity of the three-hour wait. The geography, season and population served could influence the results. While additional research in other settings might provide more definitive estimates of consultation length, the magnitude probably will not deviate much. A physician who sees 60 patients in the 480 minutes of an 8-hour day averages 8 minutes per patient, and at 100 patients per day, about 4.8 minutes. The medical student's presence may have altered the physician's performance, though we believe the direction of this would be to make the duration longer. We did not control for first versus follow-up visits, and the latter usually are longer. Finally, our analyses were based on the practice patterns of one physician. This physician was selected due to his local reputation as an excellent practitioner and family physician known to the local medical university, willingness to participate, and our belief that the selected physician was similar to other Japanese physicians. There is good reason to believe that the physician's examination style would be similar to other physicians in Japan since he trained in Japan. He is a private practitioner and faces the same productivity pressures of other private practice physicians. Although the observed physician hires others to work part-time in his office, the times observed likely would not be substantively different from those of a solo practice since he is responsible for his own patients and productivity.

Future work in additional settings and different times of year could provide more robust estimates of doctor-patient consultations in Japan. Specific comparisons of Japan with other systems that have comprehensive health care coverage would help discern more the potential pay-for-service environment of the US. Of equal interest are the implications of these shorter but more frequent visits on the doctor-patient relationship, as well as the implications for training Japan's future family physicians.

## Conclusions

The mean consultation length of 6.12 minutes is longer than the anecdotal reports of three-minute consultation lengths and is affected by variables similar to other international studies except that males have slightly longer consultation times than females. Still, 11% of visits in this study took 3 minutes or less. Although this study helps to define consultation lengths and how variables affect these times in a rural Japanese practice, there are more aspects of Japanese primary care that need to be investigated. To better understand the significance of such short consultation lengths, future research should explore consultation length in relation to continuity of care, frequency of visits, and comprehensiveness of care and compare these results with other systems with nationalized healthcare.

## Competing interests

The authors declare that they have no competing interests.

## Authors' contributions

ANW and MDF conceived and designed the study. ANW collected data while NA, AS, and MDF contributed to data analysis and interpretation. ANW and MDF drafted the manuscript while all authors critically revised and approved the final manuscript.

## Authors' information

ANW studies medicine at The Ohio State University College of Medicine:

NA serves as Data Manager, Department of Family Medicine, University of Michigan: AS serves as Biostatistician, Department of Family Medicine, University of Michigan: MA serves as Director, Amenomori Family Clinic, Shiga, Japan: MDF serves as Associate Professor, Department of Family Medicine at the University of Michigan and Director, Japanese Family Health Program at Dominos Farms Family Medicine.
